# Aging Mother–Adult Daughter Differentiation, Psychological Well-Being, and Parental Status

**DOI:** 10.3390/healthcare11131865

**Published:** 2023-06-27

**Authors:** Sesong Jeon

**Affiliations:** Major in Child & Family Studies, School of Child Studies, College of Human Ecology, Kyungpook National University, Daegu 41566, Republic of Korea; ssjeon@knu.ac.kr

**Keywords:** aging mother–adult daughter differentiation, psychological well-being, moderating effect, parental status, Actor–Partner Interdependence Model (APIM)

## Abstract

Despite the understanding that differentiation is a lifelong process crucial for psychological adaptation, there is limited knowledge regarding how parent–child differentiation in adulthood is associated with the psychological well-being of both parents and adult children. Furthermore, empirical research has yielded inconclusive results regarding whether the parental status of adult children influences the parent–child relationship. Consequently, the current study focuses on the moderating effect of adult daughters’ parental status on the association between aging mother–adult daughter differentiation and psychological well-being. The study utilized data from 167 pairs of Korean aging mothers and adult daughters to examine two main aspects: (1) the relationship between aging mother–adult daughter differentiation and psychological well-being; and (2) the moderating role of adult daughters’ parental status on the relationship between aging mother–adult daughter differentiation and psychological well-being. The findings revealed that both the differentiation of adult daughters and mothers was positively associated with their respective psychological well-being. However, no significant cross-interactional effects of aging mother–adult daughter differentiation on psychological well-being were observed. Notably, there was a positive moderating effect of the adult daughter’s parental status on the association between aging mother–adult daughter differentiation and psychological well-being for aging mothers.

## 1. Introduction

The bond between mothers and daughters has been characterized as the most intimate and robust within families [1,2]. According to Chodoro [3], as the mother identified her daughter as her expanded self, the mother saw double identification between her daughter and her mother. Due to this dual identification relationship, psychological separation is not achieved. Research has shown that the mother–daughter relationship significantly influences women’s mental well-being throughout their lives [4,5]. While scholars have examined parent–child relationships and their impact on psychological well-being in adulthood [6,7], limited knowledge exists regarding how parent–adult child differentiation influences each other’s psychological well-being. According to Bowen’s observations [8], individuals with high self-differentiation tend to experience lower levels of anxiety, while those with low self-differentiation tend to experience higher levels of anxiety. The concept of convergence stands in opposition to self-differentiation. Convergent relationships are characterized by excessive dependence, misunderstandings, and difficulties disengaging from one another. In highly fused families, it becomes challenging to distinguish one’s own identity from that of others within the family unit. The concept of family differentiation, which involves managing the balance between intimacy and individuality within a family [9], has received limited attention in the study of adult households. Korean aging mothers and their adult daughters face challenges in regulating their emotions. The Korean culture has made progress in promoting women’s education and social involvement. Although there have been shifts in gender roles and perspectives, an underlying patriarchal culture still persists, resulting in gender inequality. Within patriarchal societies, women are traditionally responsible for household duties and raising children. There is a desire for spouses who are both career-oriented and capable of managing multiple roles while maintaining a harmonious work–life balance. Mothers tend to be more involved in their children’s lives compared to fathers. This heightened involvement, along with societal pressures, leads full-time caregivers and socially active women to excessively intervene in their children’s development to prevent feelings of neglect. Despite the evolving role of women in society, their fixed role within the household, which is slow to change, contributes to an increasing burden on women to fulfill multiple roles [10]. Within this sociocultural context, it is important to examine the psychological inclination of mothers to form emotional connections with their same-sex daughters within their relationships with their children.

Despite receiving a westernized education that promotes independence and differentiation, Korean daughters continue to maintain emotional attachments to their parents even after becoming married [11]. Likewise, Korean aging mothers remain closely connected to their adult daughters [12]. The relationship between adult daughters and their mothers can have impact on the mental well-being of both parties. However, research on parent–adult child dynamics often overlooks the significance of differentiation patterns. Furthermore, studies examining the influence of parental status on psychological well-being have produced inconsistent findings [13,14]. These conflicting results emphasize the need for further empirical investigation to gain a better understanding of how parental status affects the relationship between adult children and their parents.

The objective of this study is to investigate the impact of emotional dynamics between Korean aging mothers and their adult daughters on their psychological well-being. By employing the concepts of linked lives and historical time and place within the life course perspective, the study aims to examine two main aspects: (1) the influence of parent–child differentiation and psychological well-being on the parent–child relationship, and (2) the moderating role of adult daughters’ parental status in the association between parent–child differentiation and psychological well-being.

The study will utilize the concept of linked lives, which views the family as a cohesive unit and emphasizes the shared life opportunities and development among family members [15]. Previous empirical research has consistently shown that the quality of parent–child relationships significantly impact individuals’ overall well-being [16,17]. Additionally, the study will incorporate the concept of historical time and place from the life course perspective, which recognizes the interplay between microsocial and macrosocial influences on families [15].

While differentiation is considered important for psychological adaptation throughout life [18], there is a scarcity of studies focusing on older generations [18]. Existing research on differentiation has mainly concentrated on individual psychological well-being during adolescence and early adulthood [19,20]. Moreover, limited knowledge exists regarding how mother–daughter differentiation in adulthood affects the psychological well-being of both individuals. Exploring this dynamic is particularly crucial in Korean culture, as previous studies have indicated that aging parents tend to exert excessive control over their adult children [21], while other research has shown that the adult daughters in Korea may remain excessively emotionally close to their biological parents [22]. This blurred psychological boundary may hinder appropriate independence in adulthood and potentially impact psychological well-being. To date, only two studies have investigated intergenerational self-differentiation in the mother–daughter relationship. Kim’s study [23] focused solely on the influence of mothers on daughters, neglecting the influence of daughters on mothers. Choi’s study [22] examined the connection between mother–daughter differentiation and parental role satisfaction, but it did not assess psychological well-being. To bridge this gap, the present study adopts a dyadic approach to enhance our understanding of intergenerational relationships between mothers and daughters in adulthood, especially exploring the association between mother–daughter differentiation and their psychological well-being. The association between parent–child differentiation and psychological well-being may be influenced by the parental status of adult daughters. A recurring question in intergenerational relationships is whether adult children become closer to their parents upon becoming parents themselves. However, previous studies investigating the connection between parental status and relationship quality have yielded inconsistent results [14,24]. These inconsistent findings suggest that the significance of parental status may vary across culture or society [17]. In the Korean context, parenthood has traditionally been valued both at the individual level, associated with personal happiness, and at a societal level, linked to societal contributions. However, there has been a recent increase in the proportion of individuals expressing preference for a childless family [25]. Parental status is considered one of the social structural characteristics that can associate the quality of relationships between older parents and their adult children. Hence, the present study aims to examine the moderating effect of adult daughters’ parental status on the relationship between parent–child differentiation and psychological well-being.

Based on the life course perspective, this study examined two research questions and their corresponding hypotheses:

Research Question 1. Does the aging mother–adult daughter differentiation correlate with the psychological well-being of both parties?

**Hypothesis 1.1.**  *Adult daughters with higher levels of differentiation will experience greater psychological well-being (actor effect)*.

**Hypothesis 1.2.**  *Aging mothers with higher levels of differentiation will experience greater psychological well-being (actor effect)*.

**Hypothesis 1.3.**  *Adult daughters with higher levels of differentiation will be associated with greater psychological well-being in their aging mothers (partner effect).*

**Hypothesis 1.4.**  *Aging mothers with higher levels of differentiation will be associated with greater psychological well-being in their adult daughters (partner effect)*.

Research Question 2. How does the parental status of adult daughters relate to the association between aging mother–adult daughter differentiation and psychological well-being?

**Hypothesis 2.1.**  *Being a parent will be linked to positive aging mother–adult daughter differentiation and greater psychological well-being*.

## 2. Materials and Methods

The study utilized data from 167 pairs of Korean aging mothers and their adult daughters in a dyadic format. The survey was conducted in Korea between 2014 and 2015 and encompassed inquiries about triad family relationships, especially involving adult daughters, their mothers, and their husbands. However, the primary focus of this study was solely on examining the relationship between adult daughters and their aging mothers.

### 2.1. Procedures

The data for this study were collected from the Project on Mother and Daughter Differentiation, Solidarity, and Relationship Satisfaction between Mother-in-law and Son-in-law. Convenience sampling was employed, and recruitment strategies included posting on various websites, distributing flyers at coffee shops, subway stations, and bus stations. In addition, potential participants were contacted through counseling centers, senior welfare centers, and family centers. The survey was conducted from December 2014 to February 2015, involving a total of 167 families. Although the project involved three individuals (adult daughters, their mothers, and adult daughters’ spouses) within each family unit, this study focused solely on the data obtained from adult daughters and their aging mothers.

During the data collection phase, the study included participants who met the following criteria: (1) Families residing in Daegu and North Gyeong-buk provinces, where an adult daughter and her mother live; (2) Families in which the adult daughter has been married for a minimum of one year; and (3) Families in which the aging mother possesses the necessary literacy skills to respond to the questionnaire items. Each participant was provided with a 15$ (USD) compensation for their participation in the survey. Initially, a total of 180 families took part in the study, but ultimately, 167 families successfully completed the entire research process; 13 families were excluded from the analysis for specific reasons. Among these, seven families were excluded as they resided in different provinces meaning that the mothers, who briefly visited their adult daughters’ homes on the survey date, did not meet the inclusion criteria. Additionally, six families were excluded because the adult daughters were already grandmothers.

For this study, a sample of 167 Korean aging mothers and their adult daughters residing in Daegu and Gyeong-buk provinces in the southern region of Korea was utilized. The average age of the aging mothers was 59.61 years with a standard deviation of 4.83, while the average age of the adult daughters was 34.22 years with a standard deviation of 4.83. Regarding education, more than half of the mothers had completed middle school (29.9%) or high school (33.5%). In contrast, a plurality of adult daughters had completed a four-year university degree (49.1%). In terms of employment, 37% of the mothers and 62.9% of the adult daughters had a job. The average monthly household income was lower for the mothers, at KRW 2,995,500 (USD 2668.36), compared to the adult daughters, whose average monthly income was KRW 4,739,329 (USD 4221.74). Approximately 25% of adult daughters did not have children, while around 75% reported having at least one child. A summary of the demographic information for both the mothers and adult daughters can be found in Table 1.

### 2.2. Measurement

#### 2.2.1. Psychological Well-Being

To assess the level of psychological well-being in mothers and their adult daughters, the Korean-translated version of Ryff ’s 18-item scale [26] was utilized [27]. The Psychological Well-Being Scale [26], a theoretically-grounded instrument specifically designed to measure various aspects of psychological well-being, was used as an indicator of individuals’ quality of life. This scale consists of six subscales (autonomy, self-acceptance, personal growth, purpose in life, environmental mastery, and positive relationship with others). Each subscale consists of three items, with response options ranging from 1 (strongly disagree) to 5 (strongly agree). A higher score indicates a greater level of well-being for each subscale [28]. While it is possible that psychological well-being can be characterized by a six-factor structure, the items proposed by Ryff to represent this structure lack sufficient evidence to substantiate its existence. The discrepancy could arise from shortcomings in the measurement tools, the underlying theory, or a combination of both. To address this issue, Springer et al. [29] suggested analyzing the covariance structure of the items and their associations with other variables. In light of this, they proposed two potential approaches. The first approach involves consolidating all the items into a comprehensive well-being index that encompasses various dimensions of psychological well-being. The second approach consists of merging the four overlapping subscales into a single index while treating the remaining two dimensions separately, allowing for a more detailed examination of the construct. Consequently, item-parceling was employed in the present study.

When the sample size is relatively small and fewer parameters are desired, item parcels are a preferred method of breaking down multiple item scales into two or more groups [30,31]. The use of item parcels offers several advantages to researchers, including (1) reducing sampling errors, (2) obtaining more stable parameter estimates with a more concise model, and (3) achieving better model fit [30,32].

In this study, the creation of item parcels involved several steps. Initially, exploratory factor analysis with a single factor extraction using SPSS 24.0 and principal axis method was employed. Subsequently, the items were ranked based on their factor loadings, from the highest to lowest scores. To achieve a more parsimonious model considering the six dimensions of psychological well-being, the domain-representative approach, as suggested by Little and colleagues [31], was utilized. Each parcel included multiple facets divided into item sets. For example, the first parcel consisted of autonomy 1, self-acceptance 1, personal growth 1, purpose in life 1, environmental mastery 1, and positive relationship with others 1. Similarly, the second parcel included autonomy 2, self-acceptance 2, personal growth 2, purpose in life 2, environmental mastery 2, and positive relationship with others 2. The third parcel comprised autonomy 3, self-acceptance 3, personal growth 3, purpose in life 3, environmental mastery 3, and positive relationship with others 3. Finally, the three parcels were created by calculating the average loadings of each set of items [33]. Higher scores on the parcels indicate a higher level of psychological well-being. The Cronbach’s alpha values for each subscale of psychological well-being were 0.48–0.71 for adult daughters and 0.46–0.78 for aging mothers, respectively. More detailed information is as follows: (1) autonomy (Cronbach’s α = 0.61 for adult daughters, Cronbach’s α = 0.67 for aging mothers); (2) self-acceptance (Cronbach’s α = 0.71 for adult daughters, Cronbach’s α = 0.78 for aging mothers); (3) personal growth (Cronbach’s α = 0.48 for adult daughters, Cronbach’s α = 0.57 for aging mothers); (4) purpose in life (Cronbach’s α = 0.53 for adult daughters, Cronbach’s α = 0.48 for aging mothers); (5) environmental mastery (Cronbach’s α = 0.50 for adult daughters, Cronbach’s α = 0.46 for aging mothers); (6) positive relationship with others (Cronbach’s α = 0.68 for adult daughters, Cronbach’s α = 0.60 for aging mothers).

#### 2.2.2. Aging Mother–Adult Daughter Differentiation

To assess the level of differentiation between aging mothers and their adult daughters, the Parent–Child Differentiation Scale [34] was employed. This scale, initially developed in English by researcher Chun [34] was translated into Korean and utilized in the current study. The scale consists two dimensions, namely, intimacy and individuation, each comprising 10 items. Examples of these items include statements such as, “I am satisfied with the current relationship with my mother/daughter” and “My mother/daughter does things that embarrassed me”. Participants rated their agreement with these statements on a scale ranging from 1 (strongly disagree) to 5 (strongly agree). A higher score on the intimacy subscale indicates greater connectedness between generations, while a lower score suggests disengagement or cut-off. Similarly, a higher score on the individuation subscale reflects greater separateness between generations, while a lower score indicates an enmeshed or fused relationship [8,34]. To determine the level of differentiation, Chun [34] calculated total scores for each subscale (ranging from 10 to 50) and multiplied them together, resulting in a differentiation score ranging from 100 to 2500. For instance, if someone obtained a total score of 30 on the intimacy scale and 30 on the individuation scale, their differentiation score would be 900. However, the multiplication of these total scores raised concerns about non-normality. To address this issue during the analysis, item parcels were created. First, an “a priori questionnaire construction” approach based on the nature of the scales was employed to create item parcels [31]. The parent–child differentiation scale contained both positively and negatively worded items, and, following the recommendation of Little et al. [31], the negatively worded items were reverse-coded. Exploratory factor analysis with a single-factor extraction was then conducted and items were rank-ordered based on their factor loadings. Finally, three parcels were formed by calculating the average loadings of each set of items [33].

Higher scores on the scale indicate a greater degree of differentiation in the parent–child relationships. In other words, higher scores suggest that well-differentiated parent–child dyads have a flexible and complementary relationship, while lower scores indicate that poorly differentiated dyads have a rigid or overly close relationship. The Cronbach’s alpha values for the intimacy and individuation subscales were 0.92 and 0.82 for adult daughters and 0.90 and 0.79 for mothers, respectively.

#### 2.2.3. Parental Status

The parental status of the adult daughters was assigned a code of 1 if they had their own children and 0 if they did not. Out of the total sample, there were 125 adult daughters who had children, while 42 adult daughters did not have children.

#### 2.2.4. Control Variables

While the primary focus of this study is the associations of family differentiation and psychological well-being of mothers and their adult daughters, it is important to consider that other variables may also be associated with psychological well-being. To account for these relationships, the study included control variables that served as predictors of psychological well-being. For adult daughters, the control variables included age and level of education, while for aging mothers, the control variables included age, level of education, and health status. The ages of adult daughters and aging mothers were utilized as continuous variables. Regarding the level of education, a higher academic background after graduation corresponded to a higher score. The scoring system for education level was as follows: non = 1, elementary school = 2, middle school = 3, high school = 4, two-year junior college = 5, four-year university = 6, graduate school or higher = 7. In terms of the health status of the aging mothers, a 5-point Likert scale was employed for coding, where a higher score indicated a better condition. The coding categories for health status were very poor = 1, poor = 2, adequate = 3, good = 4, and very good = 5. These control variables were taken into consideration to better understand the relationship between family differentiation and psychological well-being in both generations.

### 2.3. Analytic Plan

Before conducting the current analysis, a descriptive analysis was performed on the main scales used, including measures of central tendency (mean) and variability (standard deviation), as well as an assessment of skewness and kurtosis. More detailed information regarding these analyses can be found in Appendix A. Notably, the skewness and kurtosis values indicated that the variables utilized in this analysis adhered to the assumption of a normal distribution. Specifically, the skewness values ranged between −0.51 and 0.33, with absolute values less than 2, while the kurtosis values ranged between −0.49 and 0.66, with absolute values less than 7.

**Figure 1:**
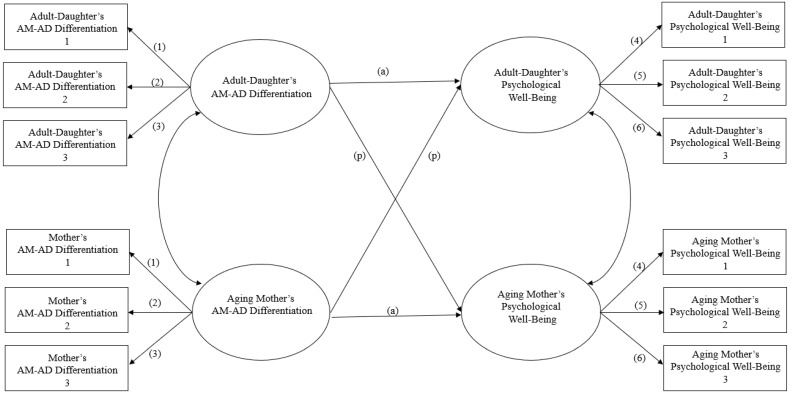
Research model. Note: (a) = actor effect; (p) = partner effect. Note: AM-AD Differentiation = Aging Mother-Adult Daughter Differentiation.

In order to investigate the potential association between aging mother–adult daughter differentiation and psychological well-being, the study employed the Actor–Partner Interdependence Model (APIM) using the statistical software M-plus version 8.0 (refer to Figure 1). The APIM analysis was chosen as it allows for the examination of both the individual and relational interdependencies within the collected pair data [35]. To explore the role of parental status in the relationship between aging mother–adult daughter differentiation and psychological well-being, the parental status of adult daughters was utilized as a moderator. The moderating effect of parental status on the association between aging mother–adult daughter differentiation and psychological well-being was examined using multiple group analysis in M-Plus.

In this study, there were no missing values for the main measurements, including psychological well-being, aging mother–adult daughter differentiation, and parental status. However, some of the control variables had missing values, specifically two missing values for the age of mothers and one missing value for the aging mother’s health status. To address these missing values, full information maximum likelihood (FIML) estimation was employed. FILM estimation allows for the use of all available information in the dataset to estimate parameters without the need to delete cases with missing values [36].

## 3. Results

### 3.1. Measurement Model

The measurement model for this study included all latent variables, allowing for free correlations between the variables. The results of the measurement model indicated a good fit to the data, with χ^2^ (88, N = 167) =110.42, *p* < 0.05, CFI = 0.98, TLI = 0.98, RMSEA = 0.04 (95% confidence interval [CI]: 0.00, 0.06), and SRMR = 0.033. A comparison of fit indices between different models is presented in Table 2. The measurement model was compared to a free model as a causal model, as well as partially constrained and fully constrained models that reflected the same structure between aging mothers and their adult daughters. In the partially constrained model, the paths involving each parceled item were constrained, while in the fully constrained model, the paths involving each parceled item, the actor effect paths, and the partner effect paths were constrained.

### 3.2. Actor-Partner Effect

The findings of the final Actor–Partner Interdependence Model (APIM) are presented in Table 3. The fully constrained model demonstrated a good fit to the data. The results indicated that daughters’ aging mother–adult daughter differentiation was positively linked to their own psychological well-being (β = 0.37, *SE* = 0.07, *p* < 0.001); similarly, their mothers’ aging mother–adult daughter differentiation was positively associated with their own psychological well-being (β = 0.43, *SE* = 0.07, *p* < 0.001). However, there were no significant partner effects of aging mother–adult daughter differentiation on psychological well-being (β = −0.02, *SE* = 0.07, *p* > 0.05 for aging mothers; β = −0.02, *SE* = 0.07, *p* > 0.05 for adult daughters). While the focus of this final model was on the relationship between aging mother–adult daughter differentiation and psychological well-being, certain control variables remained significant predictors of psychological well-being. Specifically, the age of aging mothers (β = 0.19, *SE* = 0.08, *p* < 0.05), the age of adult daughters (β = 0.20, *SE* = 0.07, *p* < 0.01), and the health status of aging mothers (β = 0.41, *SE* = 0.07, *p* < 0.001) were positively associated with their psychological well-being.

### 3.3. Moderating Effect of Parental Status

A multiple-group analysis was conducted to examine the influence of parental status on the relationship between aging mother–adult daughter differentiation and psychological well-being. Both constrained models (partially and fully constrained model) demonstrated a satisfactory fit to the data: the partially constrained model yielded χ2 (204, N = 167) = 247.08 (141.56 for the group with adult daughters who have no children; 105.52 for the group with adult daughters who have children), *p* < 0.05, CFI = 0.97, TLI = 0.97, RMSEA = 0.05 (CI: 0.02, 0.07), and SRMR = 0.08. The fully constrained model yielded χ2 (210, N = 167) = 251.15 (143.45 for the group with adult daughters who have no children; 107.70 for the group with adult daughters who have children), *p* < 0.05, CFI = 0.97, TLI = 0.97, RMSEA = 0.05 (CI: 0.02, 0.07), and SRMR = 0.08.

The results pertaining to Hypothesis 2.1 are presented in Table 4. In the partially constrained model, a moderating effect was observed between aging mothers’ differentiation and their own psychological well-being. Specifically, there was no significant difference between adult daughters who have children (β = 0.29, *SE* = 0.12, *p* < 0.05) and childless adult daughters (β = 0.54, *SE* = 0.22, *p* < 0.05) in terms of the association between aging mother–adult daughter differentiation and psychological well-being. Well-differentiated adult daughters, regardless of their parental status, exhibited higher levels of psychological well-being. However, a significant difference was observed for aging mothers. Aging mothers with adult daughters who have children displayed well-differentiated relationships, leading to greater psychological well-being (β = 0.46, *SE* = 0.12, *p* < 0.001). Conversely, aging mothers with childless adult daughters did not show a significant relationship between aging mother–adult daughter differentiation and psychological well-being (β = 0.20, *SE* = 0.25, *p* > 0.05).

However, according to the fully constrained model, no moderating effects were found between aging mother–adult daughter differentiation and psychological well-being. Regardless of the adult daughter’s parental status, both well-differentiated adult daughters and their aging mothers exhibited higher levels of psychological well-being. In the groups of adult daughters who have children, highly differentiated pairs reported greater psychological well-being (β = 0.35, *SE* = 0.07, *p* < 0.001, and β = 0.42, *SE* = 0.08, *p* < 0.001, respectively). Similarly, in the groups of adult daughters without children, well-differentiated pairs also showed higher levels of psychological well-being (β = 0.34, *SE* = 0.08, *p* < 0.001, β = 0.41, *SE* = 0.09, *p* < 0.001, respectively).

## 4. Discussion

The findings from the APIM analysis are as follows: (1) The results supported the first two hypotheses, indicating that the aging mother–adult daughter differentiation of both mothers and daughters is positively associated with their own psychological well-being. (2) However, the second two hypotheses were not supported, indicating that the aging mother–adult daughter differentiation of mothers and daughters is not positively related to the psychological well-being of the other person. (3) The results partially supported the third hypothesis, indicating that aging mothers with adult daughters who have children and maintain well-differentiated relationships experience higher level of psychological well-being compared to aging mothers with adult daughters who have no children.

These findings have three important implications. Firstly, the finding that mothers’ aging mother–adult daughter differentiation has an impact on their own psychological well-being supports Bowen’s assertation. That is, the differentiation can serve as a crucial indicator of psychological adjustment among older adults [15]. Conversely, the influence of daughter’s aging mother–adult daughter differentiation on their own psychological well-being is not surprising. This result aligns with previous research emphasizing the significance of the family of origin even after marriage. Internationally, the level of differentiation of adult children from their parents has consistently been identified as a significant predictor of their psychological well-being. For instance, differentiation from family of origin has been shown to influence marital stability [37] and marital adjustment of adult children [38]. Furthermore, a healthier experience with the family of origin has been associated with higher levels of relationship satisfaction, while poor differentiation from the family of origin has been linked to increased risk of depression among adult children [39].

Secondly, contrary to the hypotheses, it was found that aging mother–adult daughter differentiation did not have an impact on the partner’s psychological well-being. Aging mother–adult daughter differentiation may not be a significant variable for the psychological well-being of the partner in adulthood. In Korean culture, which emphasizes collectivism, individuals are encouraged to prioritize family harmony and it can be challenging to establish a strong sense of differentiation within the family unit [40]. Women, in particular, who place high value on relationships, may be more susceptible to the influence of others’ opinions and feedback [41]. However, this cultural context may not have the same effect on married daughters and their aging mothers who have already entered adulthood. Therefore, the significance of aging mother–adult daughter differentiation for them may not be as pronounced as during adolescence. Another possible interpretation is related to the educational attainment discrepancies between aging mothers and their adult daughters, which could be associated with the results. In this study, while aging mothers had lower levels of education, most adult daughters had higher levels of education. Obtaining education was less prioritized for the mothers’ generation due to the influence of a patriarchal family system [42]. Consequently, this generation of aging mothers tended to focus more on their children’s accomplishments, as reported by Chung and Chin [43]. However, in contemporary Korean culture, adult daughters have been taught to value independence and place greater emphasis on their careers and immediate family. As a result, they may not consider the psychological well-being of their original family as strongly.

Thirdly, the parental status of adult daughters acted as a moderator in the relationship between aging mother–adult daughter differentiation and psychological well-being. Although this finding does not indicate complete interdependence between them, it partially aligns with the perspective of linked lives. Parenthood of adult daughters can potentially enhance the psychological well-being of aging mothers, as they both have the shared experience of being parents. This finding suggests a connection between the level of aging mother–adult daughter differentiation and the potential provision of grandchild care in Korea. While the current study did not specifically investigate whether aging mothers were actively raising their grandchildren, it is likely that they engage in childcare duties when their daughters have children. In the Korean context, when married women receive childcare support from their mothers, it tends to strengthen their emotional connection with their mothers [44]. This emotional bond may potentially contribute to the higher reported psychological well-being of the mothers.

However, it is important to exercise caution when interpreting these findings. Since the number of dyads with adult daughters who have children (N = 125 pairs) was three times larger than the group without children (N = 42 pairs), it is necessary to consider potential errors that may arise due to the smaller sample size.

### Limitations and Future Directions

First, additional research is required to broaden the study’s scope and include a diverse range of groups, such as low-income families, as well as different geographic regions, including both urban and rural areas. The present study specifically examined adult daughter–aging mother dyads from middle-class backgrounds living in Daegu and Gyeong-buk provinces, located in the southern region of Korea. Regarding the sampling approach, the chosen sample size of 167 pairs of aging mothers and adult daughters is considered sufficient and provides a solid foundation for analysis. However, it is important to acknowledge that convenience sampling introduces a potential bias into the results since it may not fully represent the entire target population. Moreover, future research should consider the inclusion of diverse demographic characteristics. Additionally, recognizing that the psychological well-being of older adults is shaped by lifelong interaction with significant individuals [8], it would be advantageous to extend the investigation to encompass other familial relationships such as older couples and the dynamics between grandparents and grandchildren. Furthermore, it is important to explore mother–adult child dyads across different age groups. The findings of this study, which revealed no significant impact of aging mother–adult daughter differentiation on each other’s psychological well-being, suggest potential changes in the characteristics of Korean families influenced by evolving family dynamics compared to previous generations. To investigate the evolving nature of the mother–daughter relationship, future research could involve comparing the differentiation between the mother–daughter relationship during adulthood and the relationship between aging mothers and adult daughters in later life. Longitudinal studies examining the association between aging mother–adult daughter differentiation and psychological well-being would also be valuable. Additionally, it is crucial to consider other factors that may influence the psychological well-being of partners.

Although the current study accounted for certain control variables such as age, level of education, and health status, it did not include other important factors such as geographical proximity, co-residence, and the quality of the mother–child relationship during childhood. The health status of adult daughters was not considered as a control variable because it was not assessed in the original survey. Using the mother’s health status solely as a predictor for the psychological well-being of mothers limits the interpretation of the results. It would be more beneficial to incorporate the same variables for both members of the dyad to better understand and interpret the findings.

Second, careful consideration is warranted when interpreting the findings due to relatively low reliability observed in certain subscales of psychological well-being. Although an item-parceling approach was implemented to enhance the model’s accuracy by representing different domains, there may still be concerns regarding the interpretation of individual subscales. To delve deeper into the relationship between aging mother–adult daughter differentiation and each specific dimension of psychological well-being, an examination was conducted. Among the six subscales examined, only the “purpose in life” subscale exhibited significant results and demonstrated a good fit with the model. Notably, a partner effect was identified in the multigroup analysis. These findings underscore the necessity for additional empirical studies utilizing diverse approaches to validate Ryff [26]’s psychological well-being scale. From a theoretical perspective, subscales such as “autonomy” and “relationships with others” hold relevance to intergenerational dynamics. Employing a secondary factor analysis with two subscales could be a valuable approach to leverage the psychological well-being scale, and it should be considered in future research endeavors.

Lastly, it is essential to conduct further investigation into the parental status of adult children. When compared to their aging mothers, adult daughters who are childless may view motherhood differently. This divergence in viewpoints has the potential to influence both aging mother–adult daughter differentiation and their respective psychological well-being. The context of childlessness is significant, and nobody experiences it in the same manner [17]. Contrary to previous generations in which childbearing was viewed as a normal procedure following marriage, an increasing number of adult daughters in Korea are opting not to procreate [25]. Future research should account for this evolving trend and investigate the complex connection between aging mother–adult daughter differentiation and psychological well-being.

Despite the limitations outlined above, this research makes a valuable contribution to the existing body of literature concerning the association between mother–daughter differentiation and psychological well-being in adulthood. This present study explores the interdependent relationship between aging mothers and their adult daughters, which has received limited attention in Korea thus far. Additionally, it empirically investigates the concept of “linked lives” within the life course perspective by considering the parental status of adult daughters. Moreover, the distinct role of parental status for both aging mothers and their adult daughters offers valuable insights for researchers aiming to enhance the psychological well-being of older adults. 

## 5. Conclusions

This study aimed to examine the relationship between aging mother–adult daughter differentiation and their psychological well-being. Additionally, it explored the role of parental status in the relationship between aging mother–adult daughter differentiation and psychological well-being. The findings indicated that higher levels of aging mother–adult daughter differentiation were positively linked to improved psychological well-being for both parties involved. Surprisingly, no significant effects were found between partner’s aging mother–adult daughter differentiation and psychological well-being, contradicting the initial hypothesis. While Korea’s collectivist culture traditionally discourages individuals from being differentiated family members, this cultural context may not apply to adult families. Moreover, aging mothers whose adult daughters have well-differentiated relationships, particularly those with grandchildren, experience greater psychological well-being compared to those with childless adult daughters. This suggests a connection between the aging mother–adult daughter differentiation and the potential influence of raising grandchildren. Considering that married adult daughters with high aging mother–adult daughter differentiation receives parental support for childcare, the parental status of adult daughters may impact the psychological well-being of aging mothers. These findings contribute to the expanding field intergenerational relationship research.

## Figures and Tables

**Table 1:** Demographic characteristics of the participants.

	Adult Daughters (n = 167)	Aging Mothers (n = 167)
	N (%)	N (%)
Age		
• Mean	34.22	59.61
• SD	4.834	4.834
• Range	26–48	50–69
Level of education		
• None	0	5 (3.0%)
• Elementary school	0	38 (22.8%)
• Middle school	0	50 (29.9%)
• High school	18 (10.8%)	56 (33.5%)
• Two-year junior college	43 (25.7%)	7 (4.2%)
• Four-year university	82 (49.1%)	9 (5.4%)
• Graduate school more	24 (14.4%)	2 (1.2%)
Job status		
• No working	61 (36.5%)	105 (62.9%)
• Current working	105 (62.9%)	62 (37.1%)
• No response	1 (0.6%)	0
Total income (per month, Korean currency; KRW)		
• Mean	KRW 4,739,329 (USD 4221.74)	KRW 2,995,500 (USD 2668.36)
• SD	1,885,680	2,629,810
• Range	KRW 1,900,000- KRW 14,500,000 (USD 1692.50–USD 12,916.44)	KRW 400,000- KRW 25,000,000 (USD 356.32–USD 22,269.73)
Adult daughter’s parental status		
• Having children	125 (74.8%)	-
• No children	42 (25.2%)	-

**Table 2:** Comparison of fit among models.

Models	χ^2^	*Df*	RMSEA	CI	CFI	TLI	Δχ^2^	Δ*df*
Measurement Model	110.416	88	0.039	0.000–0.060	0.987	0.980		
Free Model	113.723	93	0.037	0.000–0.058	0.986	0.981		
Constrained Model (A)	124.214	97	0.041	0.013–0.061	0.982	0.977	10.491	4
Constrained Model (B)	126.457	99	0.040	0.010–0.060	0.983	0.978	12.734	6

Note. Constrained Model (A): Every parceled items paths were constrained: Constrained Model (B): The paths of actor effects and partner effects as well as the paths involving every parceled item, were constrained.

**Table 3:** Actor and partner effect of final model (fully constrained model).

Effect	Adult Daughters			Aging Mothers		
	β	B	*SE*	β	B	*SE*
Actor Effect	0.43 ***	0.27 ***	0.07	0.37 ***	0.27 ***	0.07
Partner Effect	−0.02	−0.01	0.07	−0.02	−0.01	0.07

*** *p* < 0.001. β = standardized coefficient; B = unstandardized coefficient. *SE* = Standard Error.

**Table 4:** Moderating effect of parental status.

Partially Constrained Model						
Effect	Adult Daughters			Aging Mothers		
	β	B	*SE*	β	B	*SE*
Model 1 (N = 167 pairs)						
Actor Effect	0.36 **	0.23 **	0.11	0.43 ***	0.32 ***	0.10
Partner Effect	0.10	0.06	0.11	−0.10	−0.07	0.11
Model 2 (N = 125 pairs)						
Actor Effect	0.29 *	0.18 *	0.12	0.46 ***	0.34 ***	0.12
Partner Effect	0.17	0.12	0.12	−0.10	−0.07	0.12
Model 3 (N = 42 pairs)						
Actor Effect	0.54 *	0.36 *	0.22	0.20	0.14	0.25
Partner Effect	−0.07	−0.05	0.24	−0.03	−0.02	0.24
**Fully Constrained Model**						
**Effect**	**Adult Daughters**			**Aging Mothers**		
	**β**	**B**	***SE***	**β**	**B**	***SE***
Model 1 (N = 167 pairs)						
Actor Effect	0.43 ***	0.27 ***	0.07	0.37 ***	0.27 ***	0.07
Partner Effect	−0.02	−0.01	0.07	−0.02	−0.01	0.07
Model 2 (N = 125 pairs)						
Actor Effect	0.42 ***	0.26 ***	0.08	0.3 5 ***	0.26 ***	0.07
Partner Effect	0.01	0.01	0.07	0.01	0.01	0.07
Model 3 (N = 42 pairs)						
Actor Effect	0.41 ***	0.26 ***	0.09	0.34 ***	0.26 ***	0.08
Partner Effect	0.01	0.01	0.07	0.01	0.01	0.07

* *p* < 0.05; ** *p* < 01; *** *p* < 0.001. β = standardized coefficient; B = unstandardized coefficient; *SE =* Standard Error. Note: Model 1 represents the overall group; Model 2 represents the group of adult daughters who have children; Model 3 represents the group of adult daughters who do not have children. In the partially constrained model, the paths with each parceled item were restricted. In the fully constrained model, the paths with each parceled item, as well as the paths of actor effect and partner effect, were restricted.

## Data Availability

Not applicable. The participants of this study did not give written consent for their data to be shared publicly, so due to the sensitive nature of the research supporting data is not available.

## Appendix A

**Table A1:** Correlations and descriptive analysis of the main variables.

	(1)	(2)	(3)	(4)	(5)	(6)	(7)	(8)	(9)	(10)	(11)	(12)
**(1)**	1											
**(2)**	0.87 **	1										
**(3)**	0.89 **	0.87 **	1									
**(4)**	0.60 **	0.62 **	0.57 **	1								
**(5)**	0.50 **	0.54 **	0.51 **	0.77 **	1							
**(6)**	0.54 **	0.57 **	0.56 **	0.81 **	0.78 **	1						
**(7)**	0.27 **	0.33 **	0.33 **	0.23 **	0.30 **	0.22 **	1					
**(8)**	0.40 **	0.43 **	0.42 **	0.27 **	0.41 **	0.30 **	0.73 **	1				
**(9)**	0.31 **	0.28 **	0.32 **	0.13	0.20 *	0.12	0.56 **	0.64 **	1			
**(10)**	0.18 *	0.20 *	0.21 **	0.25 **	0.25 **	0.18 *	0.26 **	0.22 **	0.16 *	1		
**(11)**	0.23 **	0.23 **	0.23 **	0.39 **	0.40 **	0.36 **	0.31 **	0.33 **	0.17 *	0.65 **	1	
**(12)**	0.21 **	0.22 **	0.23 **	0.29 **	0.34 **	0.32 **	0.32 **	0.32 **	0.26 **	0.66 **	0.64 **	1
**Mean**	3.89	3.89	3.72	3.83	3.82	3.72	3.32	3.58	3.36	3.18	3.44	3.28
**SD**	0.61	0.60	0.67	0.57	0.59	0.62	0.44	0.48	0.52	0.50	0.48	0.60
**Skewness**	−0.51	−0.31	−0.42	−0.11	−0.11	−0.10	−0.16	−0.01	−0.42	−0.10	0.33	0.32
**Kurtosis**	−0.16	−0.17	0.25	−0.49	−0.40	−0.35	0.10	−0.16	0.53	0.31	−0.23	0.66

Note. (1) Adult daughter’s differentiation item parcel1; (2) Adult daughter’s differentiation item parcel2; (3) Adult daughter’s differentiation item parcel 3; (4) Aging mother’s differentiation item parcel 1; (5) Aging mother’s differentiation item parcel 2; (6) Aging mother’s differentiation item parcel 3; (7) Aging daughter’s psychological well-being item parcel1; (8) Adult daughter’s psychological well-being item parcel2; (9) Adult daughter’s psychological well-being item parcel 3; (10) Aging mother’s psychological well-being item parcel 1; (11) Aging mother’s psychological well-being item parcel 2; (12) Aging mother’s psychological well-being item parcel3. Note: ** p* < 0.05; *** p* < 0.01.
